# AlphaVBeta3 Integrin expression within uterine endometrium in unexplained infertility: a prospective cohort study

**DOI:** 10.1186/s12905-017-0438-3

**Published:** 2017-09-26

**Authors:** Ahmed Elnaggar, Amr H. Farag, Mohamed E. Gaber, Mohamed Abdel Hafeez, Mohamed S. Ali, Alaa M. Atef

**Affiliations:** 1 Department of Obstetrics &Gynecology, Faculty of Medicine, Ain Shams University, Lotfy Elsayed Street, Abbasia, Cairo, Egypt; 2 Department of Obstetrics &Gynecology, Jersey General Hospital, The Parade, Jersey, UK

**Keywords:** Unexplained infertility, αvβ3 Integrin, Endometrial thickness, Doppler ultrasound

## Abstract

**Background:**

Implantation defect is one of these contributing factors for unexplained infertility. In the mid-luteal phase, when implantation is expected to happen, Integrins expression is remarkably increased. So, Integrins could potentially serve as markers for the frame of the window of implantation. αVβ3 integrin could have a role as a potential receptor for embryonic attachment. The aim of the current study is to investigate whether the women with unexplained infertility have a pattern of expression of endometrial αvβ3 integrin that could differ from those who have normal fertility or not.

**Method:**

Two groups of women have been included in this study. The first group was the Unexplained Infertility Group. This group included women diagnosed with unexplained primary infertility. The second group was the fertile Group, which included fertile parous women presented to the family planning clinic seeking contraception. 2D transvaginal ultrasound scan (TVS) was performed six days after detecting urinary LH surge. (TVS) was used to measure endometrial thickness, and subendometrial blood flow color Doppler Resistance Index (RI). On the same day of transvaginal ultrasound, endometrial samples were taken using the Endocell® office suction sampler for Immunohistochemistry (IHC) study using monoclonal mouse IgG antibodies to detect endometrial αvβ3 integrin.

**Results:**

Thirty-five fertile women with a diagnosis of unexplained infertility were included as a group I [Unexplained infertility Group] along with an equal number of fertile women as group II [Fertile Group]. The group of women with a diagnosis of unexplained infertility had a significantly lower αvβ3 integrin score when compared to the fertile group (median score 0, range:0–2 and median score 1, range: 1–3 and for infertile and fertile groups respectively, *P* < 0.0001). In addition, the unexplained infertility group had significantly higher subendometrial flow RI and Significantly thinner endometrial thickness.

**Conclusion:**

This study showed that Alpha v Beta 3 integrin is a significantly lower in endometrium in cases of unexplained infertility, which may suggest that underexpression of Alpha v Beta 3 integrin in human endometrium could be linked to defective uterine receptivity, and play a role as an unrecognized cause of infertility in this population of women. We need larger studies of adequate statistical power, ideally investigating more than one menstrual cycle in the same woman, to investigate the usefulness of using these molecular molecules in clinical practice.

## Background

Unexplained infertility has been defined, by the Practice Committee of the American Society of Reproductive Medicine (ASRM), as “inability of a couple to conceive for at least 12 months, with unremarkable standard infertility evaluation” ((ASRM), 2006). The prevalence of unexplained infertility is approximately estimated to be something between 15% to 30% [1]. There are various potential factors that can be responsible for unexplained infertility, and implantation defect is one of these contributing factors. Delicate interaction between the developing embryo and the receptive endometrium is required for a successful implantation process. This delicate interaction requires a harmonized dialogue between embryonic and maternal tissues [2]. Numerous factors including hormones, proteins and immunoglobulin are all orchestrated into preparing the endometrium for implantation. Expression of proteins, peptides and cytokines can be detected at luteal implantation phase where they serve as biomarkers for endometrial receptivity [3]*.* A large variety of integrins have been described within the luminal and glandular endometrial epithelium [4]. Integrins expression is characteristically increased in mid-luteal phase when implantation is expected to occur. That is why integrins are proposed to serve as markers for the frame of the window of implantation [5]. Noticeably, αVβ3 has been proposed as a potential receptor for embryonic attachment [6]. Patients with supposed implantation defects are noticed to have a higher incidence of decreased endometrial αvβ3 integrin expression [7].

The aim of the current study is to investigate whether the endometrial expression of αvβ3 integrin differs between women with unexplained infertility and women with normal fertility or not.

## Methods

The current study was conducted at Ain Shams University Maternity Hospital during the period between June 2014 and July 2015. The Ethics Research Committee, Faculty of Medicine, Ain Shams University approved the study protocol. The protocol was in agreement with the World Medical Association Declaration of Helsinki for Ethical Principles of Medical Research involving Human Subjects (last revised in Seoul, Korea, 2008). A thorough explanation of the purposes and procedures of the study has been given to all participants. Afterwards, They have been asked to sign an informed written consent. The study included two groups of women: The first group was the unexplained infertility group, including women previously diagnosed with unexplained primary infertility, and the second group was the fertile group, including fertile parous women attended the family planning clinic for the sake of contraception. Unexplained infertility was defined when a couple had a normal male partner’s semen analysis as well as documented ovulation, a normal shaped uterine cavity, and patent fallopian tubes in the female partner [1]. Semen analysis was considered normal in accordance to the WHO (2010) strict criteria [8]. Ovulation was confirmed if the mid luteal serum progesterone level was ≥3 ng/ml. Uterine cavity and fallopian tubes were evaluated by hysterosalpingogram or combined hysteroscopy/laparoscopy, as well as transvaginal ultrasound scan to ensure normal shaped cavity. We excluded all women who had any medical comorbidity (e.g. autoimmune disease or diabetes mellitus), those who have been using ovulation induction medications in the last 3 months, or those who have been pregnant before.

Women in both groups have been asked to use urinary luteinizing hormone (LH) assay to check for ovulation, using commercially available kits (Home Ovulation Kit®, ACON® Laboratories, Inc., San Diego, CA, USA). They have been asked to start using urinary LH was on cycle day 11, and to repeat it daily till detection of the LH surge. Six days after detection of the LH surge, 2D transvaginal ultrasound scan (TVS) was performed to measure endometrial thickness, and subendometrial blood flow color Doppler Resistance Index (RI). Single sonographer performed transvaginal ultrasound using Voluson 730 Expert machine (GE Healthcare Ultrasound, Milwaukee, WI, USA) equipped with 5–9-MHz transvaginal probe. The endometrial thickness was measured, with the uterus in the sagittal plane. Calipers have been placed at the highly reflective interfaces of the endometrial-myometrial junction and the maximum measurement was obtained as the endometrial thickness. This measurement included both layers of the endometrium, but excluding the surrounding low-amplitude echo layer. After obtaining endometrial thickness measurement, Color Doppler and Pulsed Doppler systems were activated for blood flow analysis. The spatial peak temporal average intensity was approximately 80 mW/cm^2^. Wall filters (50 Hz) were used to eliminate any low-frequency signals. Sub-endometrial blood vessels were usually visualized at the endometrial periphery. Sometimes they penetrated the hyper- echogenic endometrial edge or even reached the endometrial cavity. To obtain the subendometrial blood flow velocity waveforms, Doppler gate were placed the over the colored area, and the pulsed Doppler function was activated. Once 5 consecutive uniform waveforms were obtained, each demonstrating the maximum Doppler shift, the recording was considered to be satisfactory and the Resistance Index (RI) was then calculated for subendometrial vessels.

**Fig. 1:**
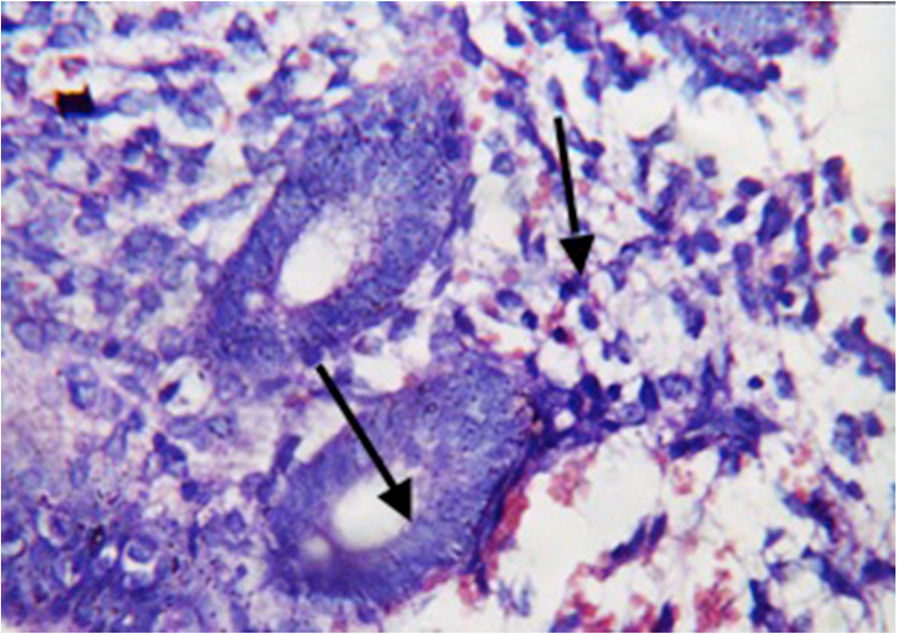
Negative controls analysed on adjacent endometrial tissue sections incubated without primary antibody. Arrows refer to glandular epithelium and endometrial stroma

**Fig. 2:**
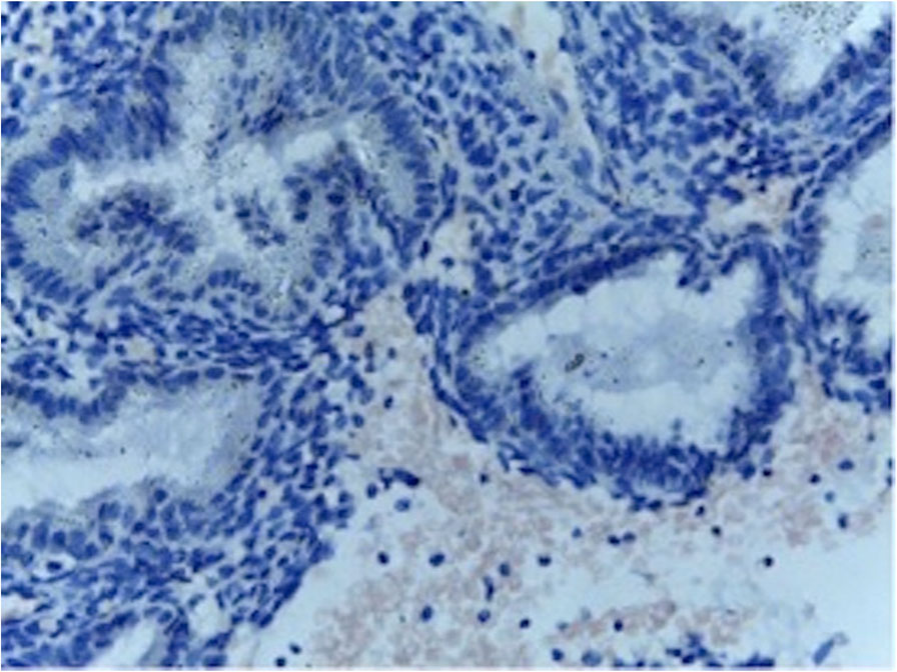
Immunostained slide with anti-avβ3 integrin, DAB chromogen, magnification x 200, score 0 in a case of unexplained infertility group

**Fig. 3:**
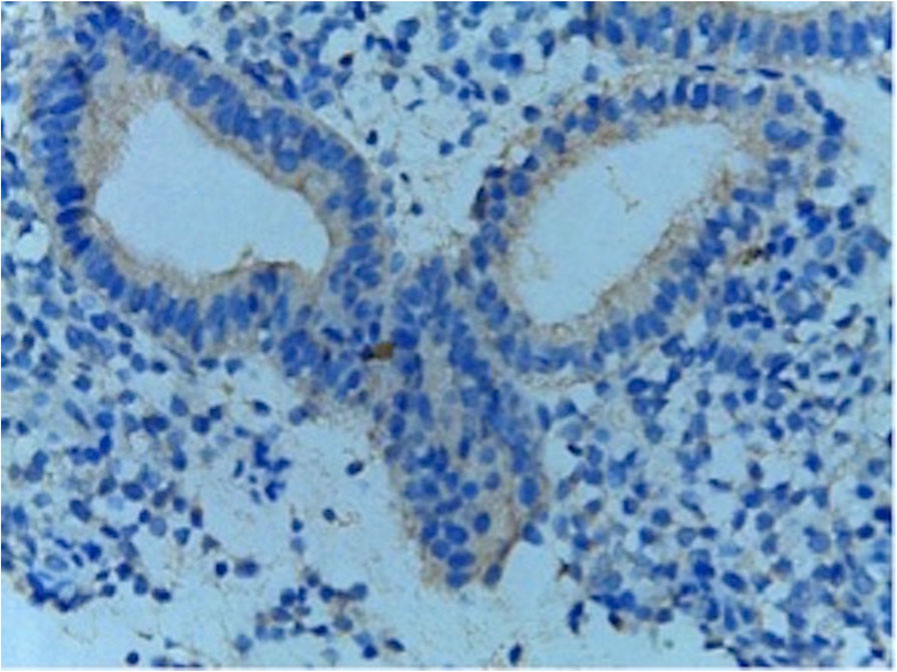
Immunostained slide with anti-avβ3 integrin, DAB chromogen, magnification x 200, score 3 in a case of fertility group. Reactivity is mainly in the galndular epithelium)

**Fig. 4:**
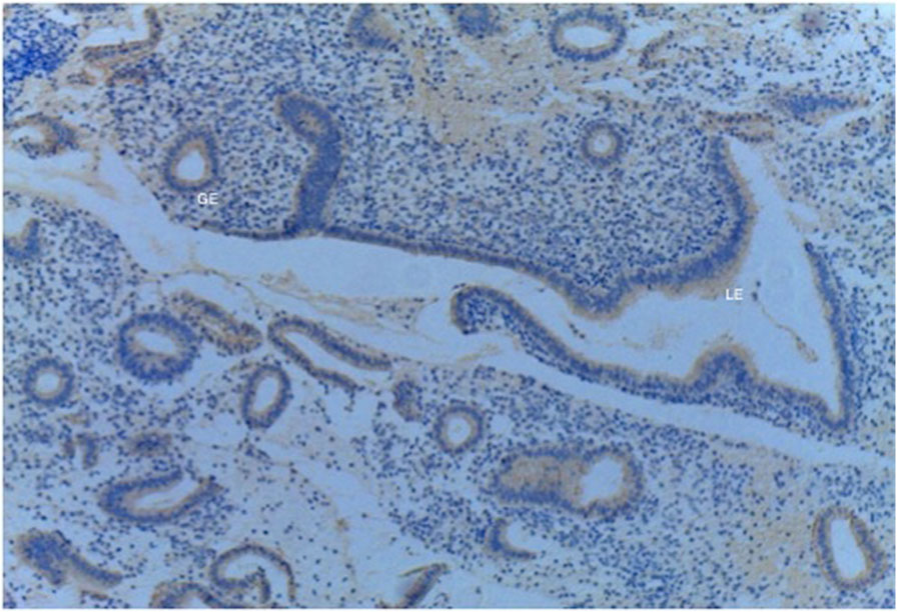
Immunostained slide with anti- vβ3 integrin, DAB chromogen, magnification x 40 (Low Power), score 3 in A case of fertility group. Reactivity could be seen in both Luminal Epithelium (LE) the Glandular Epithelium (GE)

**Fig. 5:**
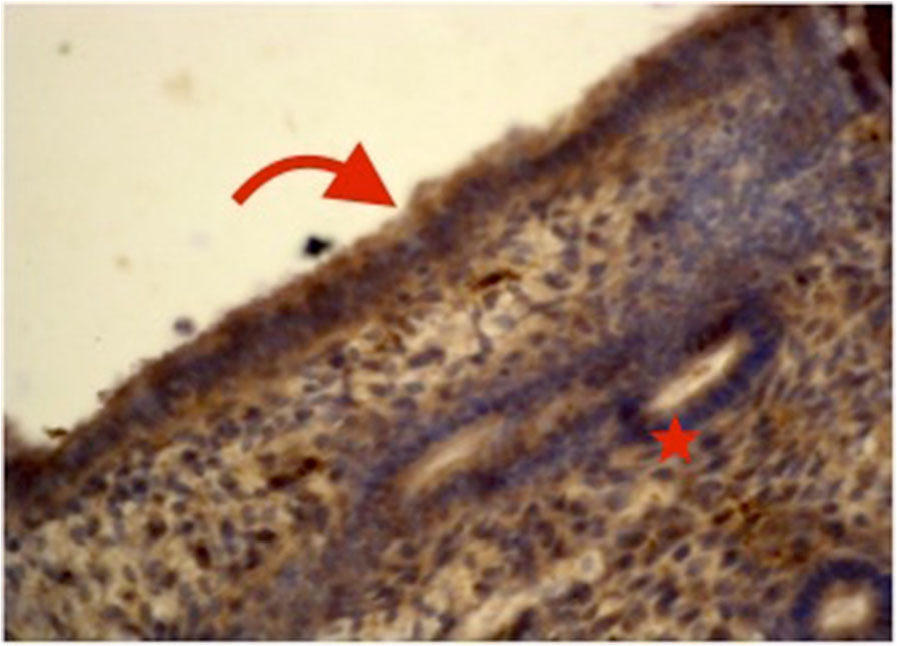
Immunostained slide with anti- vβ3 integrin, DAB chromogen, magnification x 200 (High Power), score 3 in A case of fertility group. Reactivity could be seen Mainly in Luminal Epithelium (red arrow) rather than the Glandular Epithelium (red star)

On the same day of transvaginal ultrasound, endometrial samples were taken using the Endocell® office suction sampler (Wallach Surgical Devices®, Inc., Orange, FL, USA). Endometrial Samples were fixed in 10% formalin-acetic acid in a sterile container labeled with the patient‘s study number. Samples were refrigerated at -4 °C till the time of histopathological examination. The biopsy samples were embedded in paraffin and cut into 4 μm sections. For each specimen: two paraffin sections had been prepared for routine H&E staining for secretory dating and to exclude any endometrial pathology, and another two paraffin sections had been cut on positively charged slides for immunohistochemistry (IHC) study using monoclonal mouse IgG antibodies directed against human αvβ3 integrin (Human integrin alphaV beta3 MAb (clone 23C6)®, MAB3050, R&D systems, Minneapolis, USA). Cryostat sections, 4 μm thick were prepared. These sections were placed onto salinized slides, and were fixed in cold 4% paraformaldehyde in phosphate- buffered saline (PBS) pH 7.4–7.6 for 15 min and in acetone at −20 °C for 10 min. We used the streptavidin–biotin peroxidase method for Immunoperoxidase staining (Dako Corporation, Carpinteria, CA, USA). Diaminobenzidine (DAB, Sigma Chemical Co, St Louis, MO, USA) was used as chromogen. After blocking with non-immune 4% goat serum incubated with tissues for 30 min, the primary antibody using monoclonal mouse IgG antibodies directed against human αvβ3 integrin (Human integrin alphaV beta3 MAb (clone 23C6)®, MAB3050, R&D systems, Minneapolis, USA) was added to these sections. Negative controls were analysed on adjacent sections incubated without primary antibody (Fig. 1). Two different dilutions in PBS–BSA 1% were utilized: αv 1:200, and β3 1:500. Binding was allowed to occur at 4 °C overnight. After three PBS rinses, streptavidin–biotinylated horseradish peroxidase macromolecular complex (ABC) was incubated on the sections for 30 min at room temperature before adding DAB for 7 min to complete the reaction. Samples were subsequently washed in water, counterstained with haematoxylin and mounted [9]. Positive results were detected as brownish Cytoplasmic and membranous staining. The same Pathologist performed Evaluation of staining intensity of endometrial components by a four-point semi-quantitative scoring system (0–3) as follows: (−) = no staining, (+) weak or focal staining, (++) moderate staining and (+++) strong staining [10]. This grading scale was performed by an estimate for the fraction of stained glands as follow: (−) = 0–5%, (+) = 5–25%, (++) = 25–50%, (+++) = more than 50% (Figs. 2, 3, 4 and 5).

**Fig. 6:**
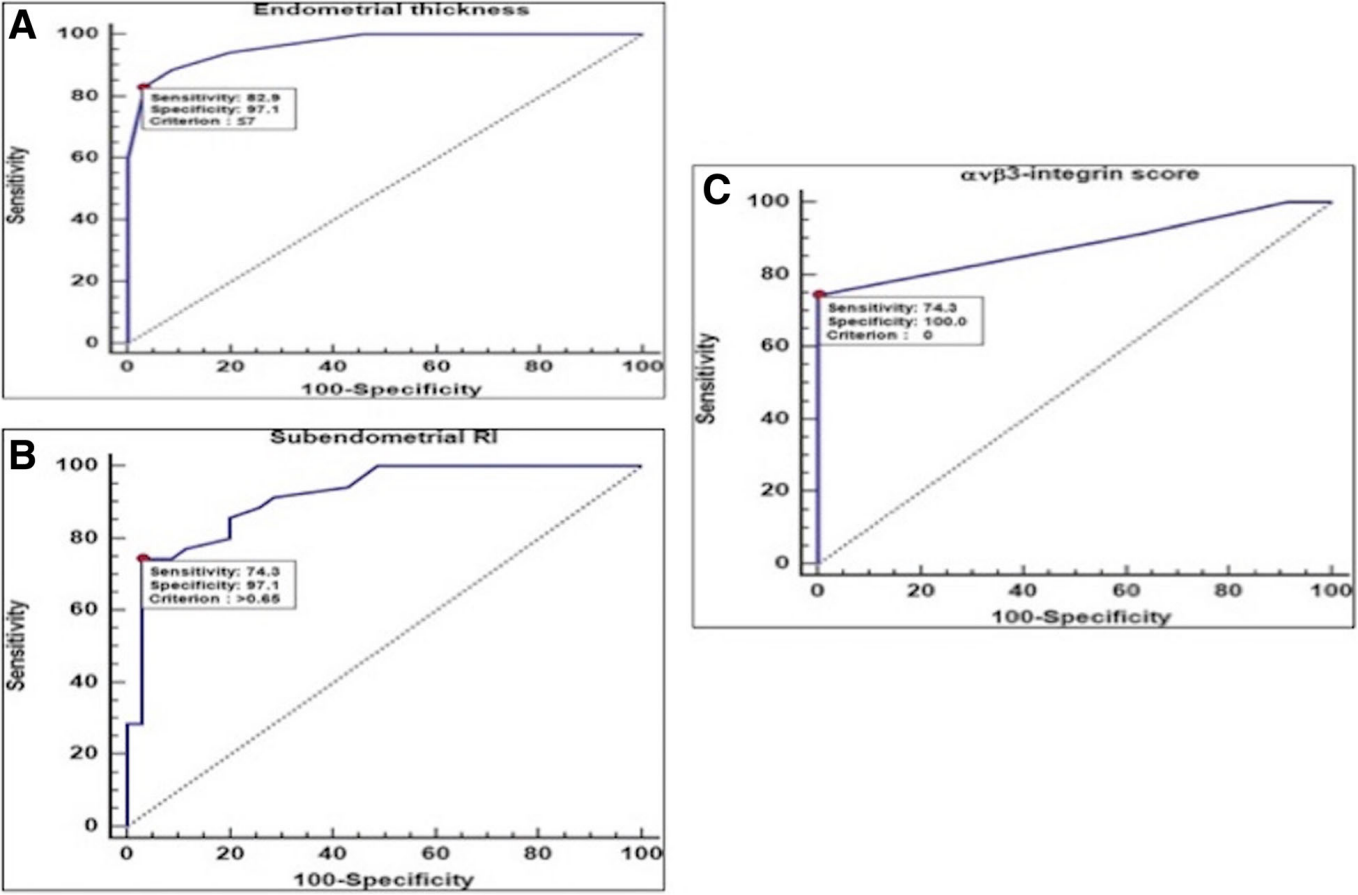
Receiver-operating characteristic (ROC) curve for the discrimination between patients with unexplained infertility and normal controls using **a**: Endometrial thickness, **b**: Subendometril RI, **c**: avβ3 integrin

### Statistical analysis

Data were analyzed using IBM© SPSS© Statistics version 22 (IBM© Corp., Armonk, NY, USA) and MedCalc© version 14 (MedCalc© Software bvba, Ostend, Belgium). Continuous numerical variables were presented as mean (SD), and discrete variables as median (interquartile range). Categorical variables were presented as number (%). Comparison of discrete numerical data was done using the unpaired t test. Discrete data were compared using the Mann-Whitney U test. Ordinal data were compared using the chi-squared test for trend.

Receiver-operating characteristic (ROC) curve analysis was used to examine the value of various biomarkers for discrimination between patients with unexplained infertility and normal controls. The DeLong method was used to compare the area under individual ROC curves (AUC). A two-sided *p*-value <0.05 was considered statistically significant.

## Results

Thirty-five fertile women were included as a group I [Unexplained Infertility Group] along with an equal number of women with a diagnosis of unexplained infertility as group II [Fertile Group]. The median (IQR) age was 29 years (24–32 years) and 28 years (25.75–32 years), respectively. The median (IQR) duration of marriage was 4 years (3–5 years) and 8 years (5–9 years), respectively. These differences were not statistically significant.

When compared to women of Fertile Group, women of the Unexplained Infertility Group had a significantly lower αvβ3 integrin score (median score 0, range:0–2 and median score 1, range: 1–3 and for infertile and fertile groups respectively, *P* < 0.0001), Significantly thinner endometrial thickness, significantly higher subendometrial flow RI (Table 1).

**Table 1:** Comparison of endometrial thickness, subendometrial Doppler resistance index, and ανβ3-integrin score in both study groups

Variable	Unexplained infertility (*N* = 35)	Control group (*N* = 35)	*p*-value
Endometrial thickness	5.4 (2.0)	11.0 (2.0)	<0.0001*
Subendometrial RI	0.71 (0.09)	0.53 (0.08)	<0.0001*
αvβ3 integrin score	0 (0–1)	1 (1–2)	<0.0001**

Data presented as mean (SD) or median (interquartile range)

*Unpaired test

**Mann- Whitney test

Receiver operator characteristics (ROC) curves were constructed for estimating the association between unexplained infertility and the measured variables in participants (Fig. 6). αvβ3 integrin, endometrial thickness, sub endometrial colored 2-D Doppler resistance index (RI) were significant predictors of unexplained infertility (Table 2). Receiver-operating characteristic (ROC) curve analysis of the endometrial thickness showed a significant value in the detection of unexplained infertility group, with the optimum criterion ≤7 mm (best cut off value) (sensitivity 82.9% and specificity 97.1%) Youden index (j) for optimum criterion 0.8, *P* < 0.001 (Fig. 3).

**Table 2:** Receiver-operating characteristic (ROC) curve analysis for the discrimination between patients with unexplained infertility and control group

ROC Index	Endometrial thickness	Subendometrial RI	ανβ3-integrin score
AUC	0.966	0.918	0.880
95% CI of AUC^Ŧ^	0.932 to 1.0	0.854 to 0.981	0.799 to 0.961
*P* value*	<0.0001	<0.0001	<0.0001
Youden index J	0.80	0.71	0.74
Cut-off criterion	≤7 mm	>0.65	Score 0
Sensitivity (%)	82.9	74.3	74.3
95% CI of sensitivity	66.4–93.4	56.7–87.5	56.7–87.5
Specificity	97.1	97.1	100.0
95% CI of Specificity	85.1–99.9	85.1–99.9	90.0–100.0
Positive predictive value (PPV) %	96.7	96.3	100.0
95% CI of PPV	82.8–99.9	81.0–99.9	86.8–100.0
Negative predictive value (NPV) %	85.0	79.1	79.5
95% CI of NPV	70.2–94.3	64.0–90.0	64.7–90.2

^Ŧ^AUC, area under the curve, PPV, positive predictive value; NPV, negative predictive value

*DeLong method

**Table 3:** Summary of studies looked at endometrial αVβ3 integrin in luteal phase of infertile women

Authors	Population	Main outcome measures	Control	Results
Lessey et al., 1995 [25]	87 Women with unexplained infertility	β3 integrin expression on cycle day 20–24	32 parous women	Reduced expression in infertile women with 26% and 39% had out-of-phase and in-phase defects, respectively
Creus et al., 2002 [18]	36 infertile patients	Endometrial αVβ3 integrin expression and pinopod formation on postovulatory day +7 to +8 and 4 days later	12 normal healthy women	No difference was found between fertile controls and infertile patients irrespective of endometria being in-phase or out-of-phase
Tei et al., 2003 [12]	57 infertile women, 22 of which with unexplained infertility	Endometrial αVβ3 expression in mid-secretory endometrium	10 fertile women	Endometrial αVβ3 expression was lower in women with unexplained infertility compared to fertile women and women with explained infertility
Ceydeli et al., 2005 [22]	33 infertile women	Mid-luteal endometrial glandular, luminal and stromal αVβ3 integrin expression during implantation window	33 fertile women	Average αVβ3 integrin expression was similar in different sites in both infertile and fertile women
Boroujerdnia& Nikbakht, 2008 [16]	30 women with unexplained infertility	Endometrial αVβ3 expression during implantation window	30 endometrial biopsies from hysterectomies with non-endometrial pathology	Endometrial glandular expression of β3 integrin was reduced significantly in infertile endometrium while no difference in stromal or blood vessel expression
Casals et al., 2008 [20]	107 infertile women	Endometrial αVβ3 integrin and osteopontin co-expression on post-ovulatory day +6 to +8, and 4 days later	12 normal healthy women	No difference regarding αVβ3 integrin and osteopontin co-expression was found between fertile controls and infertile patients irrespective of endometria being in-phase or out-of-phase
Casals et al., 2012 [17]	20 infertile patients with stage I or II endometriosis as the only detectable cause of infertility and 20 infertile patients with unexplained infertility	Endometrial expression and co-expression of osteopontin and αvβ3 integrin in postovulatory day +7 to +8 and 4 days later	20 infertile women undergoing tubal sterilization	Similar αvβ3 integrin and osteopontin expression between infertile patients with endometriosis and the two control groups

Similarly, analysis of sub endometrial colored 2-D Doppler showed that the Resistance index showed a significant value in the detection of unexplained infertility group, with the optimum criterion >0.65(best cut off value) (sensitivity 74.3%and specificity 97.1%) Youden index (j) for optimum criterion 0.71, *P* < 0.0001(Fig. 3).

Analysis of the αvβ3 integrin showed a significant value in the detection of case group, with the optimum criterion 0 (best cut off value) (sensitivity 74.3% and specificity 100%) Youden index (j) for optimum criterion 0.74, *P* < 0.0001 (Fig. 3).

## Discussion

The actual role of endometrial adhesive molecules in implantation process remains debatable and comprises an important question to modern research in the field of reproductive medicine. Gonzalez et al., [10] studied endometrial expressions of different Integrins and correlating these to various phases of menstrual cycle till Chung et al., [11] demonstrating that increased expression of integrin heterodimer αVβ3 and αVβ5, could play an importnant role in promoting endometrial receptivity for embryo implantation. Also, there has been considerable controversy concerning the correlation between endometrial thickness and the endometrial receptivity.

The bulk of information on the role of integrins in enhancing endometrial receptivity has come from studies that investigated quantitative and qualitative status of integrins within endometrium of fertile and infertile women or women with recurrent miscarriages [12]. Also, a recent systematic review showed controversial data regarding endometrial αvβ3 integrin expression in patients with increased incidence of implantation failure [13]. Hence, the current study related Sonographic-imaging data to molecular events in order to find out whether a combination of both can provide a better method to predict uterine receptivity.

Successful implantation is dependent upon the embryo being at the correct stage of development (developmental window), with the endometrium synchronously reaching the receptive stage (receptive window) [14].

Integrins are heterodimeric trans-membrane receptors that mediate cellular differentiation and adhesion, which is a crucial step in embryonic implantation [15].

Endometrial samples were obtained on Day +7 where the expression of αvβ3 integrin was evaluated using a semi-quantitative scoring system; (0 = absent, 1 = weak/focal, 2 = moderate and 3 = strong expression) [9], where it showed significantly less expression among the infertile group with a median score of zero and one for infertile and fertile groups, respectively, and also showed sensitivity 74.3% and specificity 100% when zero score was used as a cut-off value for the infertile group.

Studies have been providing variable outcomes regarding αvβ3 integrin expression and its relation to failed implantation with some trials concluding that the significantly decreased expression of endometrial αvβ3 integrin in unexplained infertility could be a reflection of its crucial role in enhancing endometrial receptivity at the implantation window [16].

On the other hand, other trials concluded that αvβ3 integrin expression in mid- or late-luteal endometrial biopsies did not correlate with the outcome for untreated infertile women [17, 18], or women undergoing assisted conception [19]. Also, it was concluded that recurrent implantation failure did not seem to be associated with abnormal endometrial integrin expression, which makes its use as a prognostic tool in subsequent IVF treatment of no value.

Casals et al. in their serial trials [17, 20, 21] studied heterogenous groups of patients with different interventions and small numbers of controls, in addition to focusing on co-expression of αvβ3 integrin and osteopontin. Also, with Creus et al., [18] the small numbers might have contributed to the different results. The contradictory results might be related to different statistical evaluation as the use of Cox regression method to compare in phase and out of phase endometrial samples [9, 19] or using different scoring system for integrin expression as HSCORE [22].

Moreover, Lessey et al., [23] found significant differences in immunostaining by utilizing different antibodies to the β3 integrin, with subsequent influence on integrin expression by the primary antibody used and Honda et al., [24] found that ligand binding might alter the conformation of some integrins and hence inhibit binding of some antibodies resulting in different outcomes. Table 3 summarizes the studies looked at the role of αvβ3 integrin in infertility problem.

In the current study, the infertile group had significantly thinner endometrium with a median of 5 mm versus 11 mm for controls. Also, ROC curve analysis of the endometrial thickness showed a best cutoff value of ≤7 mm for infertility group with sensitivity and specificity of 82.9% and 97.1%, respectively. Oliveira et al., [26] concluded that pregnancies are unlikely to happen when the endometrial thickness measured less than 7 mm.

In agreement with our study, El-Zenneni et al., [27] in their study on 50 women with unexplained infertility and 50 controls, concluded that the endometrial thickness was significantly less in patients with unexplained infertility. They found that a cut-off of ≤9 mm, would provide sensitivity and specificity of 74% and 74% respectively.

In one study, endometrial thickness has been studied in 2464 IVF cycles, where pregnancy rates varied from 29.4% among patients with an endometrial thickness of less than or equal to 6 mm, to 44.4% among patients with endometrial thickness of greater than or equal to 17 mm [28], however, a cut-off value couldn’t be suggested as ROC showed that endometrial thickness was not a good predictor of pregnancy rates. However, other studies found that an endometrial thickness as thin as 6 mm is still acceptable for a successful implantation [29]***.*** A figure of 4 mm was found in one study [30].

In the current study, there was a higher sub-endometrial flow RI among infertile group compared to fertile group with a median of 0.71 versus 0.53. The sub-endometrial colored 2-D Doppler resistance index (RI) showed a best cut-off value of >0.65 for the infertile group with sensitivity and specificity of 74.3% and 97.1%, respectively, where the Youden index for optimum criterion 0.71 was used.

Many studies have been conducted to evaluate the role of various ultrasound parameters in predicting pregnancy during stimulated IVF cycles, but no much information exists in the literature with regard to their role in women with unexplained infertility. In previous studies, it was concluded that sub-endometrial blood flow was significantly less in patients with unexplained infertility [27], as well as women with failed IVF/ICSI [31, 32].

Among the forementioned studies, this study has the merit of combining ultrasound parameters with immunohistochemical testing of early secretory endometrial αvβ3 integrin expression. Also, bias was minimized by maintaining homogeneity of the study groups and providing only one assessor for all the ultrasound scans as well as for all immunohistochemical assessments and hence avoiding inter-observer differences. Raine-Fenning et al., [33] performed endometrial and sub-endometrial serial 3D power Doppler analysis throughout the menstrual cycles of 29 women with unexplained infertility and 19 controls and concluded significantly reduced blood flow in the endometrium and subendometrium during the mid-late follicular phase and early luteal phase among infertile women. Likewise, Steer et al., 1994 found similar results in 35 women with unexplained infertility as compared to 23 controls [34] .

On the contrary, the study is limited by the small sample size. Another potential area of criticism is that the current study did not use HSCORE for assessment of immunohistochemical staining as well as correlation with other endometrial adhesive molecules. However, in the current study the main objective was to integrate ultrasound scan morphology and indices with endometrial αvβ3 integrin expression to help future studies establishing a tool or a scoring system to identify women at high risk of failed implantation or early miscarriages, but larger numbers of patients will be required as well as testing for other endometrial adhesion molecules.

## Conclusion

Alpha v Beta 3 integrin is a significantly lower in endometrium in cases of unexplained infertility, which may suggest that underexpression of Alpha v Beta 3 integrin in human endometrium could be linked to defective uterine receptivity, and play a role as an unrecognized cause of infertility in this population of women. We need larger studies of adequate statistical power, ideally investigating more than one menstrual cycle in the same woman, to investigate the usefulness of using these molecular molecules in clinical practice. This is to overcome the cyclic changes between different menstrual cycles.

## Abbreviations

AUC: Area under curve
IQR: Inter-quartile range
LH: Luteinizing Hormone
NPV: Negative predictive value
PPV: Positive predictive value
RI: Resistance index
ROC: Receiver- operating characteristic
TVS: Transvaginal ultrasound
